# Cell Proliferants

**Published:** 1914-09

**Authors:** 


**Cell Proliferants.** J. M. Fortescue-Brickdale, Bristol Medico-Chir. Jour., June, 1914, states that all the substances used to stimulate the growth of epithelium over wounds are related chemically. They are insoluble in water but more or less soluble in oils and fats, and are usually employed in 8% dilution in oil, lanoline or vaseline. They are all red in color and some are true dyes. The simplest chemically is amido-azo-toluol. If acetyl replaces a hydrogen atom in an amido-group, a light yellowish-red substance, known commercially as azodermin is produced. If both hydrogen atoms are replaced with acetyl radicles, a non-staining substance is produced known as pellidol. This, mixed with an equal quantity of an organic iodine compound is called azodolen, the iodine being antiseptic. Scarlet red replaces the two hydrogen atoms with the azo-beta-napthol radicle. It does not form a perfect solution but is best made up with equal parts of lanoline and paraffine ointment, in 5% to 10% strength. Two cases of poisoning, with dizziness, gastric pain, nausea and vomiting, not fatal, have been reported. Allantoin is a similar substance. In human urine, it seems to be merely a food derivative, passed through the body unchanged but in some animals it seems to be a normal metabolic product.

